# Non-prescriptionxx antibiotic use for people aged 15 years or older for cough in China: a community-based survey

**DOI:** 10.1186/s13756-021-00998-5

**Published:** 2021-08-30

**Authors:** Yan Luo, Xuewen Tang, Linling Ding, Zhujun Shao, Jianxing Yu, Yangqing Chen, Yang Zhou, Hanqing He, Zhiping Chen

**Affiliations:** 1 Institute for Immunization and Prevention, Zhejiang Provincial Center for Disease Control and Prevention, No. 3399 Binsheng Road, Binjiang district, Hangzhou City, 310051 Zhejiang Province China; 2 Chinese People’s Liberation Army General Hospital, Beijing, China; 3 State Key Laboratory of Infectious Disease Prevention and Control, National Institute for Communicable Disease Control and Prevention, Chinese Center for Disease Control and Prevention, Beijing, China; 4 School of Public Health, Xiamen University, Xiamen, China

**Keywords:** Non-prescription, Antibiotic use, Cough, Community-based

## Abstract

**Background:**

Non-prescription antibiotic use at community is a main driver of antimicrobial resistance. Cough is a common condition and prevalent in all townships, including China. This study aims to investigate the non-prescription antibiotic use for cough in China and explore to which extent antibiotic use knowledge was correctly instructed in communities.

**Methods:**

A probability-proportionate-to-size sampling method was adopted to survey from all 14 townships in Yiwu city, China. All participants were investigated by face-to-face interview on Portable Android Devices. The continuous variables were presented by mean and standard deviation or medium and inter-quartile range (IQR). The categorical variables were presented using percentage or constituent ratio. Chi-square test for univariate analysis and logistic regression for multivariate analysis were conducted to assess the odds ratios and 95% confidence intervals, respectively.

**Results:**

A total of 3034 respondents across all the 14 townships and the 50 natural villages/streets completed all key items of the questionnaire. Of 2400 (79.10%) respondents stated that they experienced cough in the past 12 months with the medium age of 36.5 (IQR: 26–49) and 12.21% (293/2400) respondents had the non-prescription antibiotic use behavior. Among those 293 respondents, the proportion of non-prescription antibiotic use for cough peaked at around 16% among people aged 30–39 years old. The major sources of antibiotics were pharmacy (77.70%) and/or family storage (43.92%).

**Conclusions:**

Non-prescription antibiotics use for cough is prevalent in the community, especially among people in their thirties. Strengthened drug purchase regulation and well-trained professional pharmacists would be promising alternatives to ameliorate AMR. Moreover, penetrating antibiotics knowledge to common citizens and is an urgent task to alleviate antimicrobial resistance. Therefore, proactive policies and regulations should be made to improve current situations.

**Supplementary Information:**

The online version contains supplementary material available at 10.1186/s13756-021-00998-5.

## Introduction

China is a country with a high consumption of antibiotics. According to the published data, each Chinese consumes 138 g of antibiotics per year. The quantity is 9 times higher than the United States [1]. Among the various accessibility to antibiotics, non-prescription use of antibiotics is prevalent in the community of China [2]. Non-prescription use of antibiotics increases the risk of inappropriate use, which is accelerating the process of antimicrobial resistance (AMR) and is the main driver in the development of drug-resistant pathogens [3]. AMR is more challenging in developing countries because the health system is more vulnerable [4, 5]. China, as the largest developing country, with one fifth of the world’s population, therefore, non-prescription dispensing of antibiotics at community indeed is a global issue. China also has the most rapid growth rate of AMR globally [6]. In the past decade, China launched some health reforms and policies for using antibiotics rationally [7], however, few impacts from those actions were observed [8, 9].

Cough and expectoration are also prevalent in China [10, 11]. For Chinese people aged 40 years or older of a national survey in 162 surveillance points, the prevalence rates were 15% for men and 8% for women, respectively. Past surveys showed that self-medication with antibiotics is common for people to treat self-limiting illnesses, like coughing, expectoration and upper respiratory tract infection (URTI) [12–14] in China. For example, a survey conducted by the National Medical Products Administration (NMPA) showed that 1892 of 7915 respondents (23.9%) chose to self-medication antibiotics instead of going to see a doctor when they had symptoms of a cold [12]. Previous studies [15, 16] have also shown the non-prescription antibiotic rates at community pharmacies for adult acute URTI without population-based data. In addition, the previous population-based surveys only limited to university students [14, 17] or children [18]. These studies have highlighted the seriousness of the non-prescribed and injudicious use of antibiotics, as well as high rate of antibiotic use for cough. However, there is no age-specific community-based accurate data to clarify the non-prescription frequency from a comprehensive view.

We hereby conduct a community-based survey among people aged 15 years and above to study (1) the proportion of non-prescription antibiotic use in population with respiratory disease related symptoms; (2) the sources and types of antibiotics used without prescription among people with respiratory disease related symptoms; (3) explore knowledge related to antibiotics use; and (4) factors that influenced people’s behavior of non-prescription antibiotics usage.

## Methods

### Study population

This study was carried out in Yiwu city, Zhejiang Province (approximately 1,318,600 inhabitants) from June to December in 2019. People who aged 15 years or older and had been living in Yiwu for more than 6 months were considered as source population. We selected people for the survey from all 14 townships in Yiwu.

### Definition

Cough: cough (defined as at least 3 days per week and at least 4 times per day) with or without expectoration in the past year (12 months) because of common cold or other reasons [10, 19].

Non-prescription antibiotic use: self-medication antibiotics without doctor’s prescription and get antibiotic from an unregulated supply chain.

### Questionnaire

The questionnaire used in the survey comprising 3 sections: (1) sociodemographic information; (2) non-prescription antibiotic behaviors during cough; and (3) antibiotic knowledge. People were asked to state the sources and reasons of antibiotic use when they cough and generic names of antibiotics they have used. The 9 knowledge questions related to non-prescription use and overuse of antibiotics.

### Data collection

Investigation was conducted by staffs in centers for disease control and prevention (CDC) by face-to-face communication. All investigators underwent two-round training before investigation. Probability-Proportionate-to-Size (PPS) sampling method was adopted to select people for the survey from all 14 townships in Yiwu city, then followed by a simple random sampling. The whole process of randomization and sampling is shown as a flow diagram in Additional file 1: Figure 1. The sample size is calculated by $N = \left( {\frac{{U_{\alpha /2} }}{\delta }} \right)^{2} \pi \left( {1 - \pi } \right) \times deff$ [20, 21]. We conservatively assumed that the prevalence of non-prescription use of antibiotics following cough (*π*) as 40% since there is no previous study for reference; a permissible error (*δ*) as 2.5%; a level of significance (*α*) as 5% and a design effect (*deff*) as 2. With all parameters put in, n equals to 2950. Thus, the sample size is around 3000. As for the number of clusters should be no less than 30 [21, 22], we assumed 50 clusters. A sample of 3000 divided into 50 clusters, so there were 60 individuals in each cluster. We chose 50 clusters from all these 14 townships. In each township, the number of cluster (*n*) was calculated by the following process: (1) we divided the population aged 15 or older in the Yiwu City (*N*) by 50; (2) the number of clusters (*n*) equals to the numbers containing the integer multiple of *N*/50 in the accumulative population interval of its corresponding township. Details on clusters in each township is shown in Additional file 1: Table 1. Then, we randomly chose *n* natural villages/streets as *n* clusters in corresponding township. Finally, in each cluster/natural village/street, we stratified by age group (15–19, 20–24, 25–29, 30–34, 35–39, 40–44, 45–49, 50–64 and ≥ 65 years old) and gender; 60 individuals were investigated according to the age distribution and the gender distribution. We knocked on doors according to the house number to do household investigation and chose only one person in each household. Investigation in each age group completed when its corresponding quota achieved. No-response surveys did not include in the final analysis while reasons of no response were recorded. Data collection was implemented through offline face-to-face interview system on Portable Android Device (PAD). Source data verification and data export was conducted by two investigators for quality control weekly. The data was collected between October 2019 and December 2019.

### Statistical analysis

Data analyses were done using Microsoft Excel 2010 and R v3.5.1, and *P* < 0.05 was considered statistically significant. The continuous variables were presented by mean and standard deviation (SD) or medium and inter-quartile range (IQR). The categorical variables were presented using percentage or constituent ratio. For risk factors that associated with non-prescription antibiotic behavior during cough, we used chi-square test for univariate analysis and logistic regression for multivariate analysis to assess the odds ratios (ORs) and 95% confidence intervals (CIs), respectively. Cochran-Armitage trend test was used to test the trend of age-specific frequency. Variables with statistically significant or professional significant in univariate analysis were included in the multivariate analysis. The score of antibiotic-related knowledge was calculated by simply adding the number of correct answers and each correct answer equals to one score. A score of 0–3 was considered as a low level of knowledge, 4–6 was medium and 7–9 was high.

## Results

### Sociodemographic characteristics

A total of 3051 respondents across all the 14 townships and the 50 natural villages/streets participated in the interviews. A total of 3034 (99.44%) respondents completed all key items of the questionnaire (Table 1). The spectrum of age for recruited participants spanned from 15 to 94 with the medium age of 37 (IQR: 26–49). Specially, 1819 (59.95%) lived in the urban areas and 1499 (49.41%) were male. Among the 3034 subjects, 2400 (79.10%) stated that they experienced cough in the past 12 months; the medium age was 36.5 (IQR: 26–49) (Table 1).

**Table 1:** The sociodemographic characteristics of study participants

Characteristics	Total (n = 3034)	Participants with cough (n = 2400)
	n (%)	n (%)
*Age (years)*		
15–19	210 (6.92)	179 (7.46)
20–24	420 (13.84)	327 (13.63)
25–29	416 (13.71)	329 (13.71)
30–34	318 (10.48)	251 (10.46)
35–39	312 (10.28)	239 (9.96)
40–44	314 (10.35)	250 (10.42)
45–49	313 (10.32)	241 (10.04)
50–64	417 (13.74)	333 (13.88)
≥ 65	314 (10.35)	251 (10.46)
Median (IQR)	37 (26, 49)	36.5 (26, 49)
*Residence*		
Urban	1819 (59.95)	1391 (57.96)
Rural	1215 (40.05)	1009 (42.04)
*Gender*		
Male	1499 (49.41)	1174 (48.92)
Female	1535 (50.59)	1226 (51.08)
*Occupation*		
Student	164 (5.41)	146 (6.08)
Unemployed	695 (22.91)	582 (24.25)
Business/service/food personnel	1111 (36.62)	831 (34.63)
Professional	705 (23.24)	560 (23.33)
Farmers and workers	359 (11.83)	281 (11.71)
*Education*		
Primary school and below	625 (20.60)	492 (20.50)
Middle school	1695 (55.87)	1330 (55.42)
College and above	714 (23.53)	578 (24.08)
*Child under 5 years old*		
No	1912 (63.02)	1528 (63.67)
Yes	1122 (36.98)	872 (36.33)
*Annual household income (CNY)*		
< 100,000	1789 (58.97)	1382 (57.58)
100,000–199,999	902 (29.73)	733 (30.54)
≥ 200,000	343 (11.31)	285 (11.88)
*Smoking status*		
No	1696 (55.9)	1341 (55.88)
Yes	1338 (44.10)	1059 (44.13)
*Chronic disease*		
No	2430 (80.09)	1891 (78.79)
Yes	604 (19.91)	509 (21.21)

*IQR* inter-quartile range, *CNY* Chinese Yuan

### Proportions and ratios of non-prescription antibiotics

**Fig. 1:**
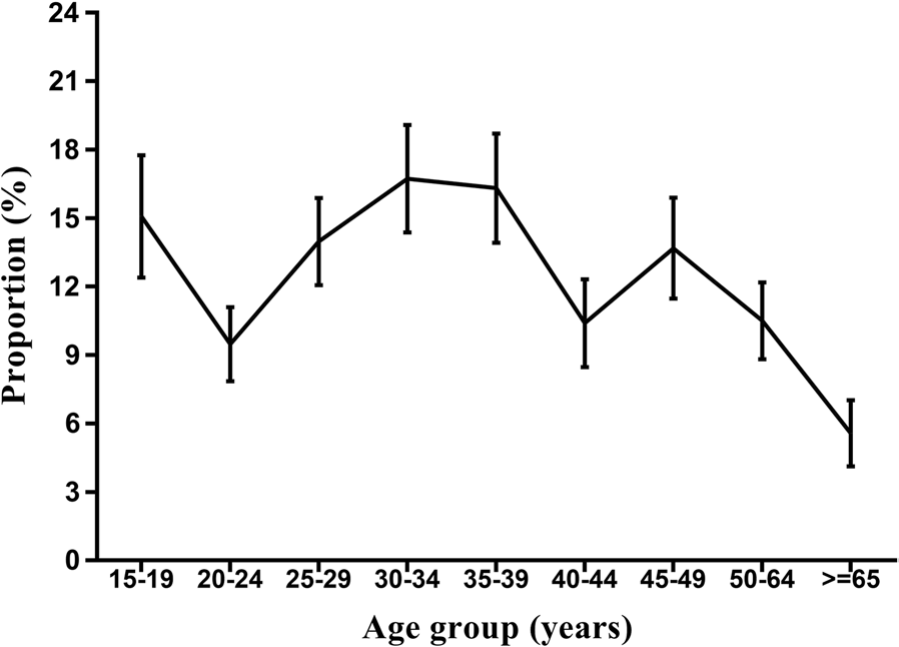
Age-specific proportions of non-prescription antibiotics use in participants for cough

Among 2400 participants who experienced cough in the past 12 months, 293 (12.21%) individuals had the non-prescription behavior. After stratified by age (Fig. 1), non-prescription proportion was ranged from 15.08% (standard error, SE: ± 2.68) in individuals aged 15–19 years, 9.48% (SE: ± 1.62) in people aged 20–24, to 13.98% (SE: ± 1.91) in individuals aged 25–29 years. Then, the proportion was peaked at 16.73% (SE: ± 2.36) among people in 30–34 years. After slightly dropped to 16.32% (SE: ± 2.39) in 35–39 years old, the proportion dramatically decreased to 10.40% (SE: ± 1.93) in 40–44 years old. Despite a few fluctuations, the overall proportion was around 10% (SE: ± 1.68) from 45 to 64 years old, then reached the lowest 5.58% (SE: ± 1.45) in people aged 65 years old or older (*Z*_trend_ = − 1.358, *P*_trend_ = 0.1743) (Fig. 1).

In 293 participants who used antibiotic without prescription when they coughed in the past year are shown in Table 2. The major sources were from pharmacy (77.70%) and family storage (43.92%), followed by last remaining antibiotic (13.18%). Only 5 (1.69%) and 3 (1.01%) individuals stated that they obtained antibiotic without prescription from friend/colleagues or online, respectively. Then we further undermine the underlying cause for non-prescription antibiotics use, 201 (67.91%) individuals considered cough was unnecessary to visit a doctor. More than half (57.09%) individuals used non-prescription antibiotic as they had antibiotic stored at home. 125 (42.23%) participants had non-prescribed use because purchasing antibiotics in pharmacy was convenient, and 102 (34.46%) participants believed that self-treating by antibiotics was effective for cough. The remaining 34 (11.60%) participants used antibiotic without prescription as they considered going to hospital was time-consuming. Only 1 (0.34%) individual non-prescribed antibiotic use because its safety (Table 2).

**Table 2:** Details of non-prescription antibiotic use in participants for cough (N = 293)

Antibiotic use	N	Ratio (%)	Ratio of options (%)
*Source*			
Pharmacy	230	77.70	55.83
Family storage	130	43.92	31.55
Last remaining antibiotic	39	13.18	9.47
Friends or colleagues	5	1.69	1.21
Online purchase	3	1.01	0.73
*Reasons for non-prescription*			
Cough is ailment and not necessary to see a doctor	201	67.91	31.36
With antibiotic at home	169	57.09	26.37
Pharmacy purchase is convenient	125	42.23	19.50
Believe self-treating by antibiotic is effective for cough	102	34.46	15.91
Too busy to see a doctor	15	5.07	2.34
The process of seeing a doctor is too long	10	3.38	1.56
Self-treatment by antibiotic is more convenient than go to hospital	9	3.04	1.40
Antibiotic is safe	1	0.34	0.16
*Antibiotic type*			
Penicillin	145	48.99	38.98
Cephalosporin	116	39.19	31.18
Don’t remember	61	20.61	16.40
Macrolides	15	5.07	4.03
Quinolones	6	2.03	1.61

### Univariable and multivariable analyses for risk factors for non-prescription antibiotic use

Table 3 shows univariate and multivariate analyses results. In multivariable analysis, families with child under 5 years old were less likely to non-prescription use of antibiotic during cough, with an OR of 0.730 (95% CI 0.552–0.965). Similarly, individuals with chronic diseases were less likely to use antibiotic without prescription when they coughed, with an OR of 0.654 (95% CI 0.441–0.969). Participants with medium knowledge of antibiotic were more likely to use antibiotic without prescription when compared to those of low knowledge, with an OR of 1.511 (95% CI: 1.122–2.034). The questions and answers to antibiotic use knowledge of 3034 respondents was shown in Additional file 1: Table 2 (Table 3).

**Table 3:** Univariate and multivariate analyses of factors associated with non-prescription antibiotic use in participants for cough (N = 2400)

Characteristics	Non-prescription antibiotic use		Univariable		Multivariable	
	Yes N (%)	No N (%)	Crude OR (95% CI)	P value	Adjusted OR (95% CI)	*P* value
*Age (years)*						
15–29	104 (12.46)	731 (87.54)	Reference	0.003	Reference	0.198
30–44	107 (14.46)	633 (85.54)	1.170 (0.891–1.536)	0.258	1.241 (0.921–1.671)	0.156
≥ 45	82 (9.94)	743 (90.06)	0.644 (0.450–0.920)	0.016	0.926 (0.575–1.491)	0.752
*Residence*						
Urban	185 (13.30)	1206 (86.70)	Reference		Reference	
Rural	108 (10.70)	901 (89.30)	0.781 (0.607–1.006)	0.056	0.799 (0.617–1.033)	0.087
*Gender*						
Male	144 (12.27)	1030 (87.73)	Reference		Reference	
Female	149 (12.15)	1077 (87.85)	0.990 (0.775–1.264)	0.933	1.040 (0.805–1.345)	0.762
*Occupation*						
Student	18 (12.33)	128 (87.67)	Reference	0.009	Reference	0.185
Unemployed	60 (10.31)	522 (89.69)	0.817 (0.466–1.432)	0.481	1.077 (0.561–2.069)	0.823
Business/service/food personnel	129 (15.52)	702 (84.48)	1.307 (0.771–2.215)	0.321	1.244 (0.697–2.223)	0.460
Professional	57 (10.18)	503 (89.82)	0.806 (0.458–1.417)	0.453	0.797 (0.427–1.486)	0.475
Farmers and workers	29 (10.32)	252 (89.68)	0.818 (0.438–1.529)	0.530	1.045 (0.518–2.105)	0.903
*Education*						
Primary school and below	39 (7.93)	453 (92.07)	Reference	0.003	Reference	0.074
Middle school	184 (13.83)	1146 (86.17)	1.865 (1.298–2.679)	0.001	1.614 (1.069–2.436)	0.023
College and above	70 (12.11)	508 (87.89)	1.601 (1.060–2.416)	0.025	1.602 (0.955–2.687)	0.074
*Child under 5 years old*						
No	196 (12.83)	1332 (87.17)	Reference		Reference	
Yes	97 (11.12)	775 (88.88)	0.851 (0.657–1.102)	0.221	0.730 (0.552–0.965)	0.027
*Household income (CNY)*						
< 100,000	167 (12.08)	1215 (87.92)	Reference	0.825	Reference	0.628
100,000–199,999	88 (12.01)	645 (87.99)	0.993 (0.754–1.307)	0.958	0.877 (0.654–1.176)	0.382
≥ 200,000	38 (13.33)	247 (86.67)	1.119 (0.767–1.633)	0.559	1.020 (0.676–1.539)	0.926
*Smoking status*						
Yes	134 (12.65)	925 (87.35)	Reference		Reference	
No	159 (11.86)	1182 (88.14)	0.929 (0.726–1.187)	0.554	0.977 (0.759–1.257)	0.855
*Chronic disease*						
No	254 (13.43)	1637 (86.57)	Reference		Reference	
Yes	39 (7.66)	470 (92.34)	0.535 (0.376–0.761)	< 0.001	0.654 (0.441–0.969)	0.034
*Knowledge score*						
0–3	87 (9.68)	812 (90.32)	Reference	0.001	Reference	0.008
4–6	134 (15.60)	725 (84.40)	1.725 (1.294–2.300)	< 0.001	1.511 (1.122–2.034)	0.007
7–9	72 (11.21)	570 (88.79)	1.179 (0.847–1.640)	0.328	1.037 (0.724–1.485)	0.842

*OR* odds ratio, *CI* confidence interval, *CNY* Chinese Yuan

## Discussion

This is the largest and the first community-based study that investigated the nonprescribed antibiotics for cough in China to date. Our findings illustrate the age-specific proportion of non-prescription antibiotic use at community for cough among residents aged 15 years or older because cough is prevalent in China [23]. We found that non-prescription antibiotic use for cough was prevalent despite of factors such as age groups, residence, gender and occupations *etc*.

The overall proportion of non-prescription is 12.21% (293/2400) for the source population, which can extrapolate to make a more accurate estimation of the non-prescription antibiotics use population for cough in national-wide. Previous studies showed the proportion was 36% in China [13] and 48.8% in a cross-sectional survey among Chinese university students [17], respectively. The inconformity between our study and past studies is due to different sampling strategy. They used the number of people who used antibiotics as denominator, while our study used the people cough in the past year as denominator. By adopting the same calculation as previous studies, the prevalence would therefore be similar to the two aforementioned studies [13, 17]. The prevalence is much higher than European countries, for instance, Sweden, Denmark, Netherlands, Austria, Belgium, Ireland and UK, for which the prevalence was approximately 3% [13, 24]. Great importance should be attached to participants in 30–39 years old, they were in the highest prevalence to used antibiotic without prescription. Some surveys reported that middle-east people aged 18–39 years with the highest prevalence while others reported a higher prevalence in people aged 40–59 years [25, 26].

Our study not only informs the high prevalence of non-prescription antibiotics used for cough, but also articulates sources, reasons, and patterns for self-medication. First, the main source of antibiotics is pharmacy, which highlights the easy access to antibiotics in communities. Chinese government launched some reforms for the purpose of using antibiotics reasonably and safely with prescription, while there were small effects on using antibiotics in health facilities. More measures for controlling antibiotics misuse should be taken. Strengthened drug purchase regulation and well-trained professional pharmacists would be promising alternatives to ameliorate AMR in developing countries [5]. Therefore, interventions for reducing non-prescription antibiotics sales in the large number of community pharmacies in China is in urgent need. Strategies involving national guidance on antibiotics for training more qualified pharmacists and delivering the WHO AWaRe antibiotic list [27] in retail shops would be effective ways to enhance pharmacists’ knowledge [28]. Second, penicillin and cephalosporin were two most common non-prescription types of antibiotics. Monitoring pharmacies using mobile technologies and/or internet to improve the regulations will be good ways for surveillance [29]. Third, participants’ knowledge on antibiotics was relatively low. Delivering pamphlets about antibiotic knowledge for community residence and use antibiotic under qualified pharmacists’ construction can be used to enhance people’s knowledge and awareness. Fourth, the residual amount of antibiotics that doctors have previously prescribed is also an important factor in misuse. Thus, establishing strict graded management system can promote rational use by limiting antibiotic prescriptions and reducing the amount of antibiotics prescribed.

Under the situation of Corona Virus Disease 2019 (COVID-19), potential threats that would affect antibiotic stewardship should not be neglected. Since the facile accessibility and little knowledge of antibiotics in China community, the irrational use of antibiotics increased for prophylaxis and self-treatment [30]. As a consequence of COVID-19, the disruption of vaccination and other health service will also increase risk of infection that ultimately leads to more prevalence non-prescription antibiotics use. Considering the COVID-19 pandemics may last for years, the high prevalence of non-prescription antibiotics use would undoubtfully challenge the stewardship system and pose threat to the antibiotic resistance [31, 32].

To conclude, PPS sampling method has strengthened the power of our study that can clarify the age-specific frequency and reasonably representative of the population in the community. Thus, providing data for China to better understand the quantity, types, and patterns of nonprescribed antibiotics used at the population, which can inform policies, regulations, and interventions to ensure that antibiotics are used appropriately. We believe that our study would be of great importance in assisting national-wide health care policy making.

This study has some limitations. First, the 9-item antibiotic use knowledge questionnaire has not been validated in previous studies. However, these 9 questions raised fundamental factors including antibiotic indications, prescription and administration principles, adverse effects and prophylactic use. Second, we have only one-site survey in Zhejiang province, more data of other provinces will be more representative. Third, PPS sampling causes regional and family clustering. However, we divided sample into 50 clusters and only investigate one person in each household. In addition, the distribution of age and gender in participants were similar to those in source population. All of these could reduce bias.

## Conclusions

In conclusion, non-prescription antibiotics use for cough is prevalent in the community. This result may reflect the real-world situation in China national-wide. Effective policies and regulations should be made to inverse this situation and efforts should be exerted to slow down the pace of AMR.

## Supplementary Information

**Additional file 1**.** Figure 1**. Process of randomization and Probability-Proportionate-to-Size (PPS) sampling.** Table 1**. Clusters in each township.** Table 2**. The antibiotic related knowledge of participants (N = 3034).

## Data Availability

The data can be available from the corresponding author upon reasonable request.

## Abbreviations

AMR: Antimicrobial resistance
URTI: Upper respiratory tract infection
CDC: Center for disease control and prevention
PPS: Probability-proportionate-to-size
PAD: Portable android device
SD: Standard deviation
IQR: Inter-quartile range
OR: Odds ratio
CI: Confidence interval
SE: Standard error
COVID-19: Corona virus disease 2019
