# Supplementary material for: Non-prescriptionxx antibiotic use for people aged 15 years or older for cough in China: a community-based survey
Source: Antimicrob Resist Infect Control. 2021 Aug 30;10:129. doi: 10.1186/s13756-021-00998-5 (PMC8404186; doi:10.1186/s13756-021-00998-5)
Supplement: Supplementary file 1 — Additional file 1. Figure 1. Process of randomization and Probability-Proportionate-to-Size (PPS) sampling. Table 1. Clusters in each township. Table 2. The antibiotic related knowledge of participants (N = 3034). [file 13756_2021_998_MOESM1_ESM.docx]

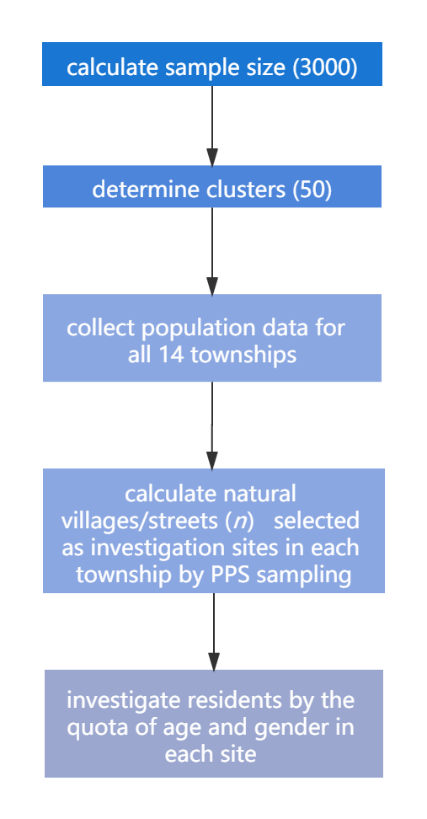


**Figure 1. Process of randomization and Probability-Proportionate-to-Size (PPS) sampling**

**Table 1. Clusters in each township**

| township | population | accumulative population | n of clusters |
| --- | --- | --- | --- |
| 1. Choucheng | 102447 | 102447 | 3 |
| 2. Choujiang | 103406 | 205853 | 3 |
| 3. Jiangdong | 145293 | 351146 | 4 |
| 4. Beiyuan | 206320 | 557466 | 6 |
| 5. Houzhai | 120236 | 677702 | 4 |
| 6. Niansanli | 82721 | 760423 | 2 |
| 7. Chengxi | 109083 | 869506 | 3 |
| 8. Futian | 171088 | 1040594 | 5 |
| 9. Suxi | 125917 | 1166511 | 4 |
| 10. Dachen | 51007 | 1217518 | 2 |
| 11. Shangxi | 109399 | 1326917 | 3 |
| 12. Futang | 197177 | 1524094 | 6 |
| 13. Chian | 53335 | 1577429 | 1 |
| 14. Titing | 110664 | 1688093 | 4 |
| Total | *N* | 1688093 | 50 |

| **Table 2. The antibiotic related knowledge of participants (N=3034)** | | | | | |
| --- | --- | --- | --- | --- | --- |
| **Questions** | **Answer N (%)** | | | | |
|  | **Completely agree** | **Somewhat agree** | **No opinion** | **Somewhat disagree** | **Completely disagree** |
| 1. antibiotic should be used as early as possible for the sake of effectiveness | 152 (5.01) | 595 (19.61) | 1103 (36.35) | 1079 (35.56) | 105 (3.46) |
| 2. antibiotic has the same effect as anti-inflammatory drug | 36 (1.19) | 871 (28.71) | 1360 (44.83) | 699 (23.04) | 68 (2.24) |
| 3. antibiotic is prescription medicine, and it should be used under doctor's prescription | 574 (18.92) | 1641 (54.09) | 703 (23.17) | 104 (3.43) | 12 (0.4) |
| 4. patients can require doctor to prescribe antibiotic because it is patients' right | 25 (0.82) | 442 (14.57) | 895 (29.5) | 1329 (43.8) | 343 (11.31) |
| 5. the higher price of antibiotic; the better of its effectiveness | 54 (1.78) | 450 (14.83) | 937 (30.88) | 1403 (46.24) | 190 (6.26) |
| 6. Intravenous infusion is more effective than oral medication | 62 (2.04) | 528 (17.4) | 954 (31.44) | 1290 (42.52) | 200 (6.59) |
| 7. broad-spectrum antibiotic can resist multiply bacteria and doctor should use broad-spectrum antibiotic first | 29 (0.96) | 353 (11.63) | 1875 (61.8) | 690 (22.74) | 87 (2.87) |
| 8. early usage of different types of antibiotics can jointly reduce antibiotic resistance | 16 (0.53) | 247 (8.14) | 1631 (53.76) | 993 (32.73) | 147 (4.85) |
| 9. antibiotic has no adverse effect and can be used prophylactically | 7 (0.23) | 199 (6.56) | 729 (24.03) | 1636 (53.92) | 463 (15.26) |
